# Incidental discovery of asymptomatic low-grade mucinous appendiceal tumor during paraumbilical hernia repair: A case report emphasizing intraoperative vigilance

**DOI:** 10.1016/j.ijscr.2024.110329

**Published:** 2024-09-21

**Authors:** Ahmad Alkheder, Ibrahim Fathallah, Abd Alrhman Alajrd, Zeina Alsodi, Majed Abdul Karim Rahal

**Affiliations:** a Department of Otorhinolaryngology, Al Mouwasat University Hospital, Faculty of Medicine, Damascus University, Damascus, Syria; b Faculty of Medicine, Syrian Private University, Damascus, Syria; c Faculty of Medicine, Damascus University, Damascus, Syria; d Faculty of Medicine, Al-Baath University, Homs, Syria; e Faculty of Medicine, Aleppo Universit, Aleppo, Syria; f Abha Private Hospital, Abha, Saudi Arabia

**Keywords:** Low-grade mucinous appendiceal tumor, Mucinous appendiceal tumor, Asymptomatic, Incidental, Paraumbilical hernia

## Abstract

**Introduction:**

Mucinous appendiceal tumor is an exceptionally rare and indolent epithelial neoplasm characterized by the production of mucin within the appendix. Here we present a rare case of a large, asymptomatic mucinous appendiceal tumor discovered incidentally during repair of a paraumbilical hernia.

**Case presentation:**

A 73-year-old man with a complex medical history presented with epigastric pain, nausea, vomiting, and constipation. Diagnosed with a strangulated paraumbilical hernia. During hernia repair surgery, a low-grade mucinous appendiceal tumor was accidentally discovered. Post-operative monitoring over 18 months, including colonoscopy and CT scans, showed no recurrence.

**Discussion:**

Appendiceal primary tumors, though rare, can originate from neuroendocrine or epithelial cells. Epithelial tumors, including mucinous adenocarcinoma, produce mucin, potentially leading to pseudomyxoma peritonei, characterized by mucinous ascites and abdominal swelling. These tumors are often incidentally discovered during surgery, as symptoms are nonspecific, resembling acute appendicitis or causing abdominal distension. Diagnosis requires histopathology, revealing mucin accumulation and irregular glandular structures. Treatment typically involves cytoreductive surgery with hyperthermic intraperitoneal chemotherapy to manage the condition effectively.

**Conclusion:**

This case emphasizes the critical need for intraoperative vigilance and histopathological analysis in detecting appendiceal mucinous tumors during abdominal surgery, ensuring accurate diagnosis and favorable outcomes.

## Highlights

• Incidental detection of a low-grade mucinous appendiceal tumor during hernia repair highlights the need for intraoperative vigilance in abdominal surgeries.
• Histopathological analysis is essential for accurate diagnosis of mucinous appendiceal tumors, potentially preventing pseudomyxoma peritonei.
• Mucinous appendiceal tumors, though rare, can present without specific symptoms, often discovered during unrelated surgical procedures.
• Prompt surgical intervention and consistent post-operative follow-up contribute to favorable outcomes.
• This case emphasizes the significance of considering appendiceal tumors in differential diagnoses, even in the absence of suggestive clinical signs.

## 1 Introduction

Mucin-secreting adenocarcinoma of the appendix, also known as mucinous appendiceal tumor, is an exceptionally rare and indolent epithelial neoplasm characterized by the production of mucin within the appendix. Its occurrence is notably infrequent, with an incidence rate of approximately 0.12 per 1,000,000 individuals [1]. This type of tumor accounts for a mere 0.2–0.3 % of all appendectomy specimens [2]. Histologically, 65 % of these tumors are neuroendocrine in nature, while only around 20 % are classified as adenocarcinomas, which include mucinous, nonmucinous, and signet ring cell subtypes. Mucinous appendiceal tumors have the potential to induce a condition known as pseudomyxoma peritonei, marked by the accumulation of gelatinous mucin (mucinous ascites) in the peritoneal cavity. Typically, appendiceal malignancies are incidentally discovered, as patients often do not exhibit any symptoms suggestive of an underlying neoplasm [3]. However, in cases involving pseudomyxoma peritonei, patients may present with increased abdominal girth. Additionally, some patients may be initially diagnosed with acute appendicitis, with the malignant nature of the tumor only being identified upon subsequent histopathological analysis [3]. In this report, we describe a rare instance of a large, asymptomatic low-grade appendiceal mucinous tumor, which was unexpectedly identified during the surgical repair of a paraumbilical hernia in a male patient.

This work is also reported in line with SCARE criteria which helped to improve the transparency and quality of this case report [4].

## 2 Case presentation

**Fig. 1:**
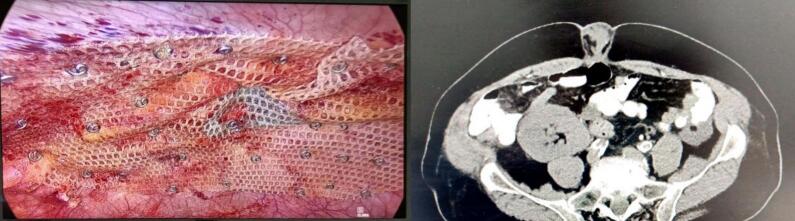
CT scan, axial plane, where the strangulated paraumbilical hernia appears, and the endoscopic repair site.

**Fig. 2:**
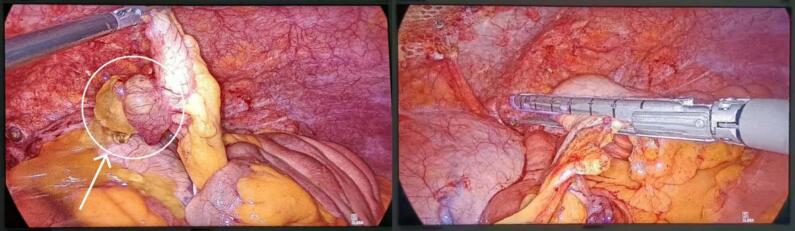
The tumor mass appears at the expense of the appendix, and view during appendectomy.

**Fig. 3:**
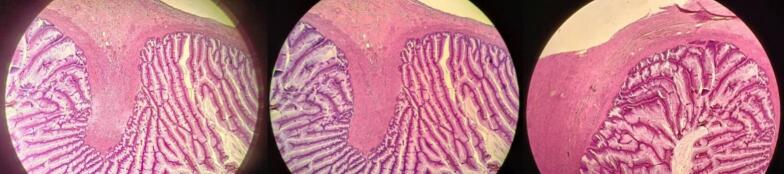
Histological study: where it appears low grade mucinous appendiceal mucinous, with mucocele, micro perforation, local extra appendiceal acellular mucinous deposition and focal serositis.

A 73-year-old man presented with a two-day history of moderate epigastric pain, accompanied by nausea, vomiting, and constipation. The pain extended to his back. His medical history includes diabetes mellitus, hypertension, a kidney transplant, coronary artery bypass graft surgery, inguinal hernia, and a right above-knee amputation. Physical examinations of the head, neck, chest, cardiovascular system, and pelvis were unremarkable. However, the abdominal examination revealed tenderness in the epigastric region, along with periumbilical distention and erythema. Based on these clinical findings, a preoperative diagnosis of a strangulated paraumbilical hernia was established. The patient subsequently underwent hernia repair, and during the repair of an endoscopic hernia (Fig. 1), a mass was observed at the expense of the appendix (Fig. 2). The appendix was excised and submitted for histological analysis. The pathology report identified a low-grade mucinous appendiceal tumor, characterized by a mucocele, microperforation, localized extra-appendiceal acellular mucin deposition, and focal serositis (Fig. 3). The patient was followed up for one year, during which a colonoscopy and CT scan were performed with no evidence of recurrence. The patient did not require any additional treatment.

## 3 Discussion

Primary appendiceal cancers are exceptionally uncommon, representing less than 0.5 % of all malignant tumors in the gastrointestinal tract. These malignancies are identified in only 0.7 % to 1.4 % of appendectomy specimens, highlighting their rarity among digestive system cancers. Despite its status as a small, vestigial organ, the appendix can develop a range of histological cancer types. These include adenocarcinomas further categorized into colonic adenocarcinoma, mucinous adenocarcinoma, and signet ring cell carcinoma as well as appendiceal neuroendocrine carcinomas. Some cases involve mixed tumors containing both of these elements, including goblet cells [12,13].

Appendiceal primary tumors are uncommon and can originate from two primary sources: neuroendocrine cells, which are nonepithelial, and epithelial cells, which line the appendix and produce mucin, a gel-like substance that helps protect the appendix, stomach, and intestines [5]. When these mucin-producing cells spread into the peritoneal cavity, they can lead to the accumulation of mucin within the abdomen, a condition known as pseudomyxoma peritonei. This causes mucinous ascites, leading to abdominal swelling, and it may be the only symptom [5]. Other epithelial tumors include goblet cell adenocarcinoma, signet ring cell adenocarcinoma, and colonic-type adenocarcinoma (nonmucinous) [5]. Mucinous adenocarcinoma of the appendix, a very rare and slow-growing tumor, secretes mucin and has an incidence rate of approximately 0.12 cases per 1,000,000 individuals [1]. It is found in about 0.2–0.3 % of all appendectomy specimens [5]. In 2010, the World Health Organization classified mucinous neoplasms into three categories based on several factors, including the presence of mucin outside cells, the degree of invasion, survival rates, and the presence or absence of pseudomyxoma peritonei [6]. Typically, these tumors are discovered incidentally during surgery or histopathological examination, as they often present with nonspecific symptoms. Initially, patients may have no symptoms or may show signs resembling acute appendicitis, such as abdominal pain and fever. However, in asymptomatic patients, the spread of mucin-secreting cells beyond the appendix can lead to mucin accumulation in the peritoneal cavity, resulting in abdominal distension, early satiety, dyspepsia, and stress-induced anorexia [5]. Metastasis outside the peritoneal cavity is extremely rare [7]. Radiological imaging, including ultrasound and CT scans, may detect a cystic dilation of the appendix, but histopathology is required for a definitive diagnosis, revealing abundant extracellular mucin and irregular glands within. Other histological findings may include loss of the lamina propria, hypertrophy of the muscularis mucosae, calcifications, and fat around the appendix [9]. The primary treatment is cytoreductive surgery combined with hyperthermic intraperitoneal chemotherapy [8].

Given that the likelihood of lymph node metastasis in well-differentiated, localized appendiceal tumors is below 2 %, most existing surgical literature indicates that a simple appendectomy is generally adequate for tumors confined to local disease. However, a right hemicolectomy may be warranted in cases where tumor margins remain involved post-appendectomy or when certain risk factors are present. These factors include tumor extension into the peri-appendiceal region, a tumor size of 2 cm or larger, high-grade histology, or invasion through the muscularis propria. Pahlavan et al. have similarly recommended right hemicolectomy based on criteria such as (a) poor cellular differentiation, (b) elevated mitotic activity, (c) involvement at the base of the appendix, (d) lymph node metastasis, and (e) tumor size exceeding 2 cm [10,11].

## 4 Conclusion

This case underscores the importance of considering appendiceal mucinous tumors in patients undergoing abdominal surgery, even in the absence of specific symptoms. The incidental discovery of a low-grade mucinous appendiceal tumor during hernia repair highlights the need for careful intraoperative evaluation. Histopathological analysis remains crucial for accurate diagnosis, given the tumor's potential to cause pseudomyxoma peritonei. This case also emphasizes the favorable prognosis associated with prompt surgical intervention and vigilant follow-up, as demonstrated by the absence of recurrence.

## Consent

Written informed consent was obtained from the patient for publication of this case report and accompanying images. A copy of the written consent is available for review by the Editor-in-Chief of this journal on request.

## Ethical approval

Ethics clearance was not necessary since the University (Damascus University) waives ethics approval for publication of case reports involving no patients' images, and the case report is not containing any personal information. The ethical approval is obligatory for research that involve human or animal experiments.

## Funding

N/A. We received no funding in any form.

## Author contribution

**Ahmad Alkheder and Zeina Alsodi:** Validation, Writing – review & editing, Visualization, Methodology, Software, Writing – original draft, Formal analysis. **Ibrahim Fathallah and Majed Abdul Karim Rahal**: Validation, Writing – review & editing, Visualization, Methodology, Software, Writing – original draft, Formal analysis. **Abd Alrhman Alajrd**: Validation, Formal analysis, Writing – review & editing.

## Guarantor

Ahmad Alkheder

## Research registration number

This case report is not a first time of reporting, new device or surgical technique. So I would not need a Research Registry Unique identifying number (UIN).

## Conflict of interest statement

The authors disclose no conflicts.

## Data Availability

All data are available from the corresponding author on reasonable request. The case has not been presented at a conference or regional meeting.
